# Langerhans Cell Histiocytosis Associated With Chronic Myeloid Leukemia: A Pediatric Case Report

**DOI:** 10.1002/cnr2.70582

**Published:** 2026-05-20

**Authors:** Atbin Latifi, Sina Yousefian

**Affiliations:** ^1^ School of Medicine Arak University of Medical Sciences Arak Iran; ^2^ Student Research Committee Arak University of Medical Sciences Arak Iran

**Keywords:** BCR‐ABL positive, child, chronic, histiocytosis, inflammation, leukemia, mitogen‐activated protein kinase 1, myelogenous, neoplasms, second primary

## Abstract

**Background:**

Langerhans cell histiocytosis (LCH) is a rare clonal neoplasm driven by activating mutations in the MAPK pathway, leading to accumulation of pathological Langerhans cells in various tissues. LCH is increasingly associated with secondary malignancies, including leukemias, lymphomas, and solid tumors. However, chronic myeloid leukemia (CML) has not previously been reported in association with LCH. Here, we describe a rare case of CML arising in the setting of active LCH in a pediatric patient.

**Case:**

A 15‐year‐old boy was initially diagnosed at age 11 with single‐system multifocal skeletal LCH. He received 12 months of vinblastine and prednisolone per the LCH‐III protocol but failed to achieve remission and was lost to follow‐up. Two years later, he re‐presented with fever, bone pain, leukocytosis, anemia, and thrombocytosis. Bone marrow analysis was consistent with CML and PCR confirmed *BCR::ABL1* p210‐positive CML. PET and MRI demonstrated concurrent active LCH lesions. The patient was treated with imatinib and cladribine, achieving complete hematologic and molecular remission of both diseases within 7 months.

**Conclusion:**

This rare association of CML with LCH expands the recognized spectrum of LCH‐associated malignancies. It underscores the importance of long‐term surveillance in LCH patients, with particular vigilance for secondary malignancies. It also highlights the need for further research into possible biological relationships between histiocytic and myeloid neoplasms.

## 1 Introduction

Langerhans cell histiocytosis (LCH) is a rare clonal neoplastic disorder of the monocyte–macrophage system characterized by the accumulation of pathological Langerhans cells (CD1a^+^/CD207^+^ dendritic cells), forming granulomatous lesions across various organs [1]. LCH is primarily driven by activating mutations in the mitogen‐activated protein kinase (MAPK) pathway, most commonly the *BRAF V600E* mutation [2]. These alterations result in sustained MEK/ERK signaling that promotes LCH cell survival, tissue accumulation [3], and recruitment of inflammatory immune infiltrates at lesion sites, thereby contributing to both local and systemic inflammation [4].

Clinically, LCH is heterogeneous and may affect nearly any organ. The skeleton and skin are most frequently involved, with bone lesions occurring in up to 80% of cases [2]. Based on disease extent, LCH is classified into single‐system (unifocal, multifocal, pulmonary, or central nervous system [CNS]) and multisystem disease [3]. Multisystem LCH is further categorized based on risk‐organ involvement (liver, spleen, and bone marrow) into risk‐organ‐positive or ‐negative subtypes [1].

Beyond its primary manifestations, LCH is increasingly associated with secondary malignancies, including leukemias, lymphomas, and solid tumors, with acute leukemias representing the predominant hematologic category [5]. The distribution of associated malignancies shows an age‐related pattern: hematologic neoplasms predominate in pediatric patients, whereas in adults solid tumors and lymphomas are slightly more frequent [5, 6]. Overall, hematologic neoplasms account for approximately half of associated malignancies in children and up to one‐third in adults [5, 7].

Within the myeloid spectrum, acute myeloid leukemia (AML) represents the most frequent association, followed by myelodysplastic syndromes (MDS) and chronic myelomonocytic leukemia (CMML). Classical myeloproliferative neoplasms (MPNs) remain distinctly uncommon [5, 7], and chronic myeloid leukemia (CML) has not been previously reported in association with LCH.

CML is a clonal myeloproliferative neoplasm characterized by uncontrolled expansion of myeloid cells at various stages of maturation [8]. Although predominantly a disease of adulthood, it represents the most common chronic myeloproliferative neoplasm in children [9]. Clinically, CML often presents with significant leukocytosis and thrombocytosis and progresses through three phases based on blast percentage: chronic (< 10%), accelerated (10%–19%), and blast crisis (≥ 20%) [9]. The defining molecular hallmark of CML is the Philadelphia chromosome, a reciprocal translocation between chromosomes 9 and 22 that generates the *BCR::ABL1* oncogene [8].

Here, we report a rare pediatric case of CML arising in the setting of active LCH, adding to the spectrum of LCH‐associated malignancies.

## 2 Case

We report the case of a 15‐year‐old boy who first presented at age 11 with a four‐week history of frontal skull deformity, intermittent fever, and lower back pain. His medical and family histories were unremarkable. On examination, he was febrile, and a tender, palpable abnormality was noted over the frontal bone.

**FIGURE 1:**
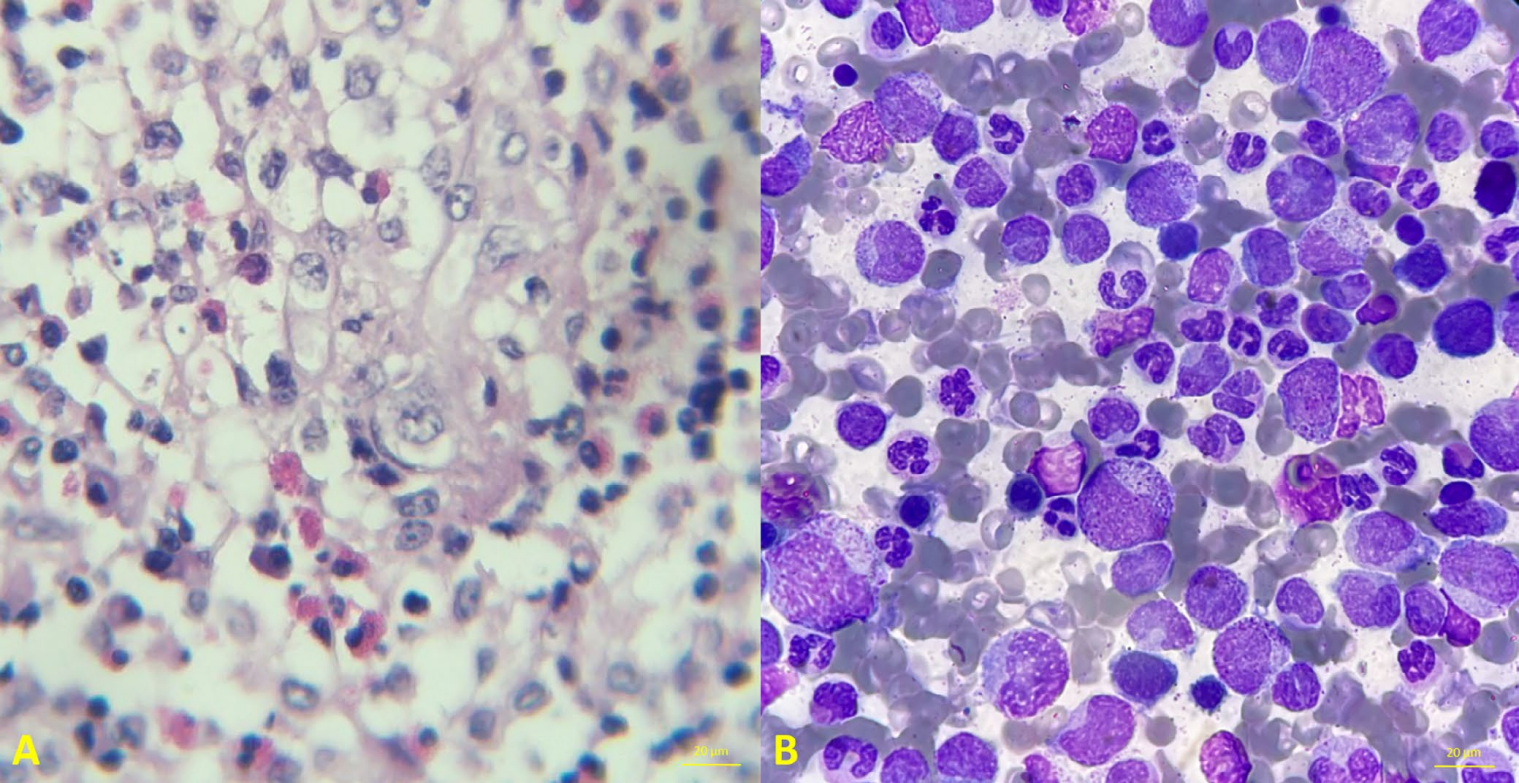
Histopathological findings of LCH and bone marrow features of CML. (A) Hematoxylin and eosin section of the LCH lesion at original magnification ×400, showing a polymorphic infiltrate composed of medium‐to‐large Langerhans cells with pale eosinophilic cytoplasm and irregular, grooved nuclei (coffee‐bean nuclei), admixed with eosinophils and lymphocytes. Multinucleated forms and nuclear pseudoinclusions are present, with scattered apoptotic bodies and karyorrhectic debris. (B) Bone marrow smear at original magnification ×1000 (oil immersion), demonstrating marked granulocytic predominance with left‐shifted maturation, increased basophils, relative erythroid suppression, and blasts < 5%, consistent with CML.

Skull X‐ray revealed two osteolytic lesions in the frontal and occipital bones. Subsequent MRI confirmed a 19 × 8 mm occipital lesion and a smaller 6 × 2 mm lesion in the left frontal bone (Table 1). Suspecting LCH, both lesions were surgically excised, followed by cranioplasty per single‐system skeletal LCH guidelines [10]. Histopathology was consistent with LCH (Figure 1A), and immunohistochemistry confirmed CD1a and CD207 positivity.

**TABLE 1:** Chronological summary of clinical, imaging, laboratory, and treatment milestones.

Date/stage	Imaging/clinical events	Laboratory results	Intervention/medication
Initial presentation (2020)	**Symptoms:** Skull deformity, intermittent fevers, lower back pain **MRI:** Osteolytic lesions in occipital (19 × 8 mm) and frontal (6 × 2 mm) bones; Suspected LCH	—	Biopsy, curettage, and cranioplasty
Diagnostic workup	**Skeletal survey:** Multifocal bone lesions (T7, T9, S1, right femoral head, left humeral head, right SI joint) No CNS or visceral involvement	**IHC:** CD1a^+^/CD207^+^ **Bone marrow:** no infiltration (CD1a−/CD207−) **Extra‐skeletal evaluation**: unremarkable	Initiated vinblastine + prednisolone (LCH‐III protocol)
Week 6 (mid‐induction)	**Bone scan:** Reduced activity (skull, T9); increased uptake (T7, femoral head, SI joint); new lesions in fifth and sixth left ribs	—	Continued vinblastine + prednisolone
Week 12 (end‐induction)	**Bone scan:** Near‐complete resolution (skull, SI joint); other sites stable	—	Switched to maintenance therapy (vinblastine + prednisolone q3w)
Month 6 (3 months of maintenance)	**Bone scan:** Resolution of most lesions; decreased uptake in rib lesions	—	Continued maintenance
Month 12 (9 months of maintenance)	**Bone scan:** Disease progression: increased uptake in occipital and rib lesions; new lesion in left eighth rib	**Targeted RT‐PCR:** *BRAF* V600E−, *NRAS*−, *KRAS*−	Planned cladribine ± cytarabine (not initiated); patient lost to follow‐up
Follow‐up interruption (approximately 2 years)	—	—	No treatment received
Re‐presentation (December 2024)	**Symptoms**: fever, malaise, bone pain; **Bone marrow**: hypercellular with granulocytic predominance and left shift; blasts < 5%; increased myeloid‐to‐erythroid ratio; basophilia; increased megakaryocytes **Skeletal survey and CT:** progression of frontal bone lesion. **PET:** no uptake in prior sites; mild uptake in left tibia and frontal bone **MRI:** left tibial lesion (T1 hypointense, T2 hyperintense)	**WBC**: 102000, **Hemoglobin**: 6.5, **MCV**: 83, **Platelets**: 1430000 **Differential:** **Basophils**: 11%, **Neutrophils**: 40%, **Band cells**: 8%, **Myelocytes**: 12%, **Metamyelocytes**: 8%, **Lymphocytes**: 12%, **Monocytes**: 3%, **Eosinophils**: 7% *BCR::ABL1* PCR (p210): positive	Initiated imatinib (340 mg/m^2^) and cladribine (5 mg/m^2^ × 5 days every 3 weeks)
1 month on imatinib and cladribine	Achieved complete hematologic and major molecular response.	*BCR::ABL1* PCR: 1.8% (MR1.0) **WBC**: 7400, **Hemoglobin**: 11.5, **MCV**: 85, **Platelets**: 340000 **Differential:** **Basophils**: 1%, **Neutrophils**: 57%, **Band cells**: 3%, **Lymphocytes**: 35%, **Monocytes**: 5%, **Eosinophils**: 2%	Continued imatinib and cladribine
3 months on imatinib and cladribine	Deep molecular response achieved.	*BCR::ABL1* PCR: 0.002% (MR4.5)	Continued imatinib and cladribine
6 months on imatinib and cladribine	—	*BCR::ABL1* PCR: undetectable (MR5)	Continued imatinib and cladribine
7 months on imatinib (post 6 cycles of cladribine)	**MRI**: resolution of tibial lesion. **PET**: no FDG uptake in tibia, frontal bone, or other regions	—	Cladribine discontinued; continued imatinib monotherapy

*Note:* Chronological summary of clinical presentation, imaging findings, laboratory investigations, and treatment interventions from initial LCH diagnosis through subsequent development and management of CML.

Abbreviations: CML, chronic myeloid leukemia; CNS, central nervous system; FDG, Fluorodeoxyglucose; IHC, immunohistochemistry; LCH, Langerhans cell histiocytosis; MCV, mean corpuscular volume; MR, molecular response; MRI, magnetic resonance imaging; PCR, polymerase chain reaction; PET, positron emission tomography; SI, sacroiliac; WBC, white blood cell count.

To assess disease extent, a skeletal survey and spinal MRI were performed, revealing additional osteolytic lesions in T7, T9, S1, right femoral head, left humeral head, and right sacroiliac joint. Bone marrow evaluation showed no histiocytic infiltration. Extra‐skeletal evaluation revealed no involvement of the liver, spleen, lungs, CNS, or skin.

In the absence of risk organ involvement, a diagnosis of single‐system multifocal skeletal LCH was established, and the patient began induction therapy per the LCH‐III protocol [10], receiving vinblastine and oral prednisolone. After 6 weeks, bone scans revealed a mixed response, with decreased activity in some lesions, increased uptake in others, and emergence of new lesions in the left fifth and sixth ribs (Table 1). As a result, induction therapy was extended for an additional 6 weeks.

Follow‐up scan after the second cycle demonstrated near‐complete resolution of the skull and sacroiliac lesions, with stable disease elsewhere and no new lesions. The patient proceeded to maintenance therapy per LCH‐III protocol, receiving vinblastine and prednisolone for 5 days every 3 weeks. After 3 months, a bone scan showed marked improvement, including resolution of most lesions and decreased activity in the remainder (Table 1). Maintenance therapy was continued for an additional 6 months.

At the six‐month follow‐up, imaging indicated disease progression, with increased uptake in some lesions and a new lesion in the left eighth rib (Table 1). Molecular testing for common resistance‐associated mutations (*BRAF* V600E, *NRAS*, and *KRAS* mutations) was performed using real‐time polymerase chain reaction (RT‐PCR), and no pathogenic variants were detected. Comprehensive MAPK‐pathway mutational profiling could not be performed because of financial constraints, limiting characterization of potential driver alterations. Vinblastine/prednisolone was discontinued, and treatment with cladribine (± cytarabine) was planned but ultimately not initiated due to parental refusal. The patient was subsequently lost to follow‐up.

After approximately 2 years without follow‐up, the patient re‐presented with a two‐month history of intermittent fever, malaise, and bone pain. Laboratory evaluation revealed leukocytosis, anemia, and thrombocytosis (Table 1). Bone marrow aspiration and biopsy revealed a markedly hypercellular marrow with granulocytic predominance and left‐shifted myelopoiesis. Blasts comprised less than 5% of nucleated cells. There was a markedly increased myeloid‐to‐erythroid ratio with relative erythroid suppression, increased basophils, and increased megakaryocytes, findings consistent with CML (Figure 1B). The diagnosis was confirmed by PCR detection of the BCR::ABL1 p210 transcript. Bone marrow immunostaining was negative for CD1a/CD207.

**FIGURE 2:**
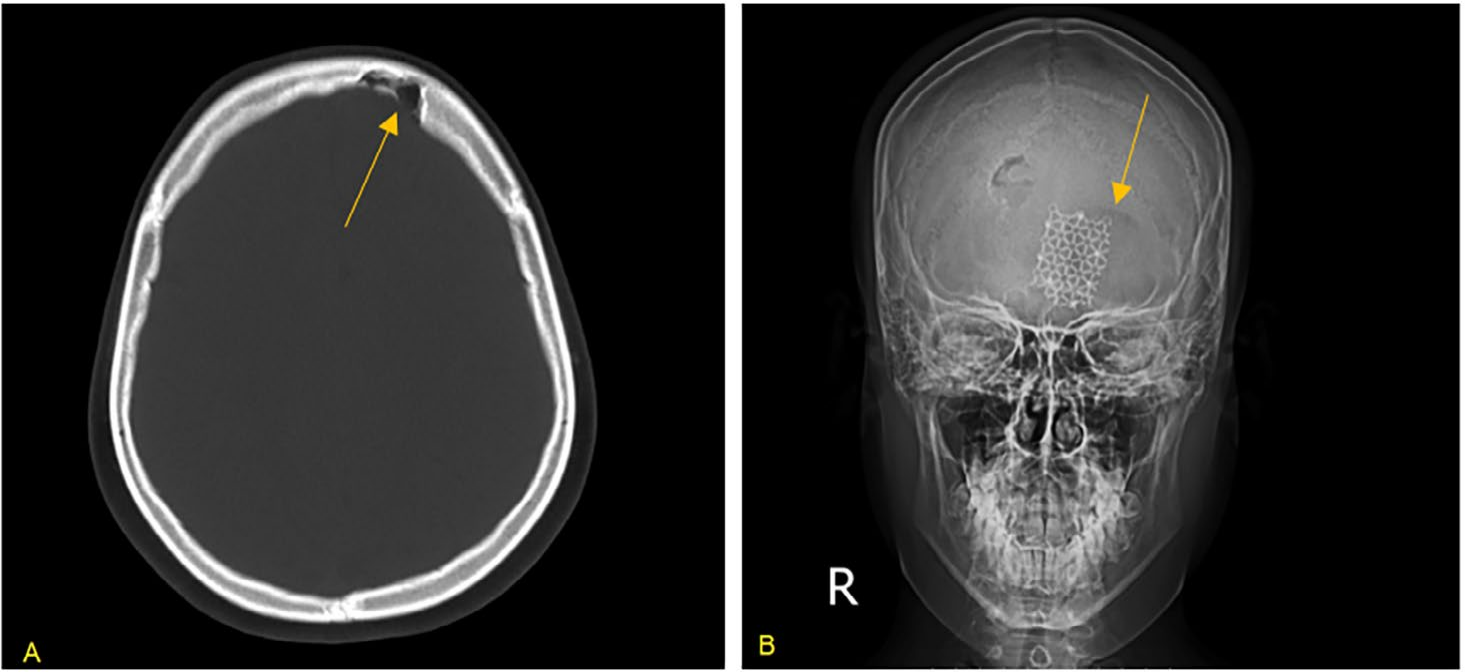
Skull imaging at the time of re‐presentation. (A) Axial CT scan showing changes at the margin of the left frontal bone resection site (arrow), suggesting local progression. (B) Frontal skull X‐ray demonstrating lucency surrounding the cranioplasty mesh (arrow), consistent with recurrence at the surgical margin.

**FIGURE 3:**
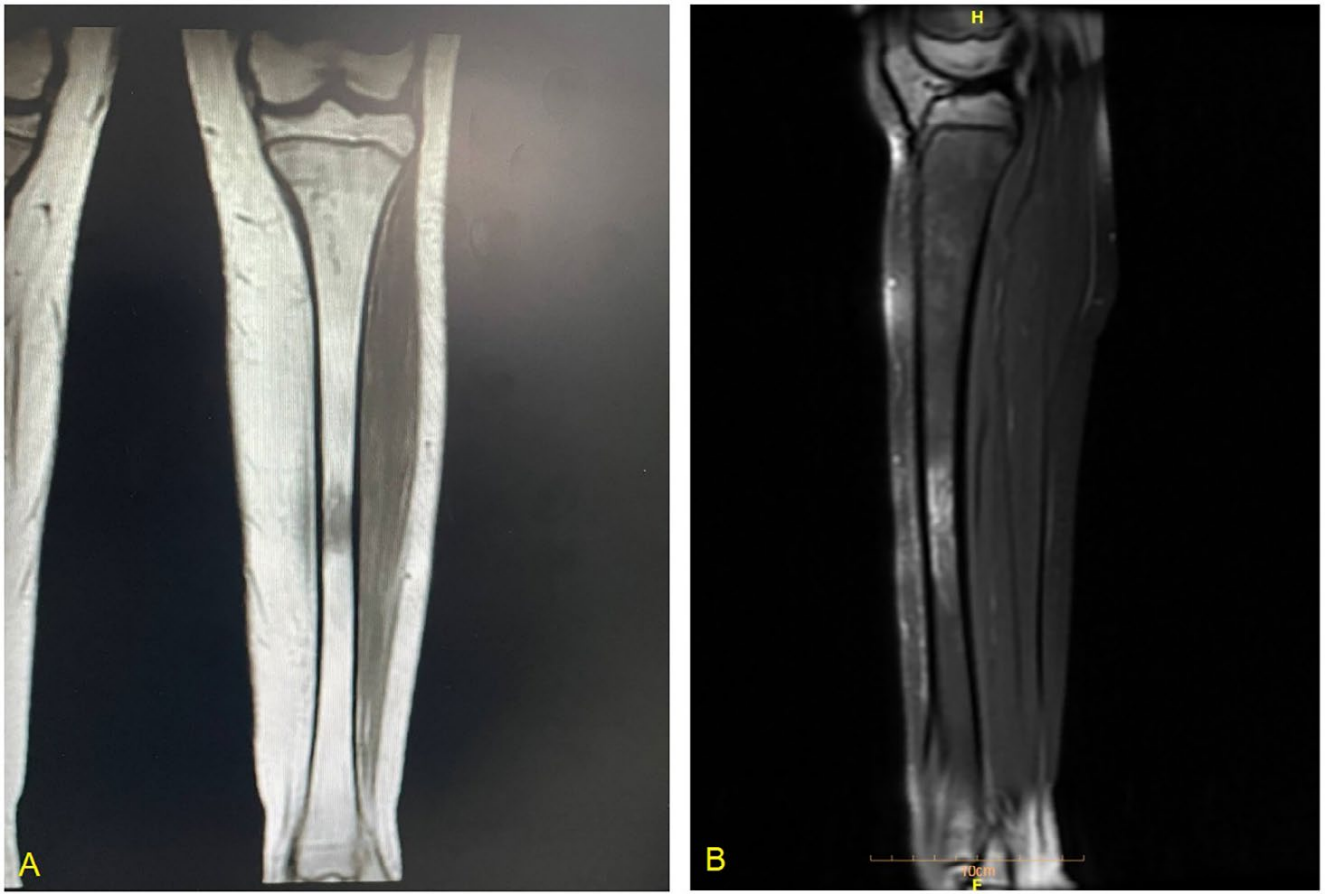
MRI of the left tibia demonstrating an active LCH‐associated osteolytic lesion. Coronal MRI of the left tibia showing (A) a well‐demarcated hypointense lesion on T1‐weighted imaging and (B) corresponding hyperintense signal on T2‐weighted imaging, consistent with an active osteolytic lesion in LCH.

Given the LCH history, a skeletal survey and CT were conducted, revealing progression at the left frontal bone resection margin (Figure 2). Positron emission tomography (PET) showed mild fluorodeoxyglucose (FDG) uptake in the mid‐left tibia and left frontal bone, with no metabolic activity at previously involved sites. MRI of the left tibia demonstrated T1 hypointensity and T2 hyperintensity (Figure 3A,B), consistent with an active lesion in the diaphysis. Imaging of the brain, lungs, and abdomen was unremarkable, with no evidence of other organ involvement.

The patient was initiated on combination therapy with imatinib (340 mg/m^2^/day) and cladribine (5 mg/m^2^/day for five consecutive days, every 21 days). Within 1 month, he achieved complete hematologic and major molecular response (MR1.0), followed by a deep molecular response (MR4.5) at 3 months, and undetectable BCR::ABL1 transcripts (MR5) by 6 months. Exact *BCR::ABL1* PCR values are detailed in Table 1.

Following six cycles of cladribine, MRI of the left tibia showed normalized signal intensity, and PET confirmed no FDG uptake at previously active sites. Cladribine was subsequently discontinued, and the patient remains on imatinib monotherapy with ongoing monitoring.

Therapy was well tolerated without adverse events, and adherence was confirmed through routine monitoring. The patient's guardians expressed satisfaction with the outcome. Informed consent for publication was obtained from the guardians, with all identifying details anonymized in accordance with ethical standards.

## 3 Discussion

Secondary malignancies occur in association with LCH more frequently than expected by chance [6]. The heterogeneity in timing and tumor type suggests multiple underlying mechanisms rather than a single pathway [6]. We report a rare case of CML arising in association with LCH, adding to the spectrum of reported LCH‐associated myeloid neoplasms.

### 3.1 Clonal Relationships in LCH‐Associated Malignancies

Molecular studies have demonstrated that LCH and associated malignancies can share a common clonal origin in a subset of patients [6]. Identical driver alterations have been reported across LCH and diverse neoplasms, including the *BRAF* V600E mutation in LCH associated with CMML [11], Hodgkin lymphoma, and hairy cell leukemia [12]; *MAP2K1* alterations in mixed histiocytosis evolving to AML [13]; recurrent mutations involving *ASXL1*, *IDH2*, *STAG2*, or *KRAS* in LCH‐AML pairs [12, 14]; and *JAK2* V617F in LCH‐PMF [15]. These observations support models of branching clonal evolution from an early mutated precursor.

In our patient, this model remains biologically plausible given the myeloid origin of LCH [4], but it could not be evaluated because *BCR::ABL1* testing was not performed on LCH tissue and molecular characterization was limited to targeted hotspot assays. Consequently, whether the two diseases reflect clonal relatedness, branching evolution from a common progenitor, or independent events cannot be determined. Nevertheless, this case documents a rare clinical association between LCH and *BCR::ABL1*‐positive CML.

Besides clonal relatedness, alternative biological models may also be considered, although none can be confirmed or excluded given the limited molecular data.

### 3.2 MAPK/ERK and Shared Signaling Pathways

The MAPK/ERK pathway—central to LCH pathogenesis—is also activated in CML downstream of the BCR::ABL1 oncoprotein [16]. Both diseases share additional signaling pathways (JAK/STAT, PI3K/AKT, NF‐κB, RAC) [1, 16] that in LCH may be activated by somatic mutations or cytokine stimulation [1], while in CML they function downstream of BCR::ABL1 [16]. Although these pathways are not independently sufficient to initiate CML, their persistent activation in LCH could theoretically establish a permissive environment that indirectly predisposes to leukemogenesis [17]. Cross‐talk between MAPK‐driven signals in LCH and *BCR::ABL1*‐associated pathways might further enhance survival signaling in early CML cells. In this patient, prolonged active LCH may have sustained such aberrant signaling, potentially contributing to a permissive setting for subsequent CML development, though this remains speculative.

### 3.3 Role of Inflammation

LCH is characterized by a systemic inflammatory state [18] with sustained cytokine dysregulation [3] and a self‐reinforcing immune microenvironment driven by interactions between LCH cells and surrounding immune infiltrates [5]. This chronic inflammation extends beyond local lesions to create systemic effects that promote oncogenesis by inducing replicative stress, impairing DNA repair, and disrupting normal hematopoiesis, thereby creating conditions permissive for secondary genetic events [19]. This concept is supported by frequent LCH‐lymphoma associations, often diagnosed concurrently, which have been interpreted as reflecting an inflammation‐driven or reactive process [18]. Our patient's prolonged active LCH may have sustained such systemic inflammation, potentially contributing to a permissive context for later CML development, though causality cannot be established.

### 3.4 Systemic Pro‐Tumorigenic Environment

An alternative explanation is that the coexistence of LCH and CML reflects a shared underlying predisposition. The frequent association of LCH with diverse secondary malignancies has led to the hypothesis of a systemic tumor‐promoting milieu characterized by chronic cytokine activation, growth factor signaling, and genomic instability [18]. In this framework, LCH and secondary neoplasms may represent parallel outcomes of an underlying dysregulated biological environment rather than sequential events. This hypothesis remains biologically plausible in our patient, though comprehensive germline testing was not performed to confirm or exclude underlying predisposition.

### 3.5 Therapy‐Related Leukemogenesis

Therapy‐related leukemogenesis represents another mechanism underlying LCH‐associated malignancies [20]. Exposure to cytotoxic agents—particularly alkylating agents, topoisomerase inhibitors, or radiation—can induce genomic instability leading to secondary neoplasms [21]. Etoposide, frequently used in pediatric oncology protocols [22], has been historically associated with secondary leukemias following LCH treatment due to its leukemogenic potential [23]. However, therapy‐related CML is exceedingly rare [21], and our patient received only vinblastine and prednisolone—agents not associated with secondary leukemogenesis—making a treatment‐induced CML in this case highly unlikely.

## 4 Limitations

Several limitations restrict interpretation. First, molecular evaluation of LCH was limited to targeted RT‐PCR for the *BRAF* V600E mutation and hotspot *NRAS/KRAS* variants; comprehensive MAPK‐pathway profiling was not performed, and therefore the potential contribution of unidentified MAPK‐pathway alterations to leukemogenesis cannot be excluded.

Second, *BCR::ABL1* testing was not performed on LCH tissue. At the time of initial diagnosis, this analysis was not clinically indicated, and at re‐presentation, repeat biopsy of skeletal lesions was not undertaken, as disease recurrence was established based on prior histologic confirmation and compatible imaging findings, and repeat biopsy is not routinely required in this setting. Therefore, assessment of *BCR::ABL1* in LCH tissue was not feasible.

In addition, the therapeutic contribution of imatinib to the observed improvement in LCH lesions cannot be determined. As the patient received combination therapy with imatinib and cladribine, and *BCR::ABL1* status in LCH tissue was not assessed, it remains unclear whether imatinib exerted any direct effect on LCH or whether the response was attributable to cytotoxic therapy. Although imatinib has been reported to show activity in selected LCH cases [24, 25], available evidence is limited and heterogeneous; therefore, any potential role of imatinib in LCH in this case remains uncertain.

Consequently, potential shared driver mutations, cooperating alterations, or shared clonal origin could not be assessed. Whether LCH and CML arose independently, evolved from a common precursor, or reflected branching clonal evolution cannot be determined. Although the temporal sequence raises the possibility of a biological relationship, coincidence cannot be excluded, and any mechanistic link remains speculative without definitive paired molecular data.

## 5 Conclusion

We report a rare case of CML arising in a pediatric patient with active LCH, thereby expanding the recognized spectrum of LCH‐associated malignancies. This observation highlights the importance of sustained hematologic surveillance in individuals with LCH, with heightened vigilance for secondary malignancies. Future investigations incorporating comprehensive molecular analyses of similar cases will be essential to better define possible biological relationships, and experimental studies may further clarify whether immune and signaling alterations in LCH are associated with leukemogenic processes.

## Author Contributions

**Atbin Latifi:** conceptualization, investigation, writing – original draft, methodology, validation, visualization, writing – review and editing, formal analysis, project administration, supervision, data curation, resources. **Sina Yousefian:** resources, data curation, software, formal analysis, writing – review and editing, visualization, validation, methodology, writing – original draft, investigation, conceptualization, project administration.

## Funding

The authors have nothing to report.

## Ethics Statement

The study was approved by Arak University of Medical Sciences Ethics Committee (date: 05/04/2024, number: IR.ARAKMU.REC.1404.054).

## Consent

Written informed consent was obtained from the patient's parents for publication of this case report and any accompanying images.

## Conflicts of Interest

The authors declare no conflicts of interest.

## Data Availability

The data that support the findings of this study are available from the corresponding author upon reasonable request.
